# Pretreatment with Shuanghe-Tang Extract Attenuates Postischemic Brain Injury and Edema in a Mouse Model of Stroke: An Analysis of Medicinal Herbs Listed in *Dongui Bogam*

**DOI:** 10.1155/2018/2479602

**Published:** 2018-02-11

**Authors:** Min Jae Kim, Seo-Yeon Lee, Ji Young Hwang, Hyunha Kim, Ki-Tae Ha, Byung Tae Choi, Jin Ung Baek, Hwa Kyoung Shin

**Affiliations:** ^1^Department of Korean Medical Science, School of Korean Medicine, Pusan National University, Yangsan, Gyeongnam 50612, Republic of Korea; ^2^Korean Medical Science Research Center for Healthy Aging, Pusan National University, Yangsan, Gyeongnam 50612, Republic of Korea; ^3^Graduate Training Program of Korean Medicine for Healthy Aging, Pusan National University, Yangsan, Gyeongnam 50612, Republic of Korea; ^4^Division of Humanities and Social Medicine, School of Korean Medicine, Pusan National University, Yangsan, Gyeongnam 50612, Republic of Korea

## Abstract

**Aim:**

Although stroke is among the leading causes of death and long-term disability, there are few effective treatments for limiting the severity of neurological sequelae. We evaluated the effects of 29 medicinal herbs listed in the *Pung* chapter of the 17th century Korean medical text *Dongui Bogam* on stroke symptoms in a mouse model of cerebral ischemia.

**Methods:**

Focal cerebral ischemia was induced via photothrombosis. Infarct volume, brain edema, and neurological deficits were evaluated. Immunofluorescence staining for tight junction proteins and aquaporin 4 (AQP4) was performed following ischemic injury.

**Results:**

Based on our initial findings, we examined the effects of two prescriptions in which the candidate herbs comprised more than 60% of the total formula: Shuanghe-tang and Zengsunsiwu-tang. Pretreatment with Shuanghe-tang significantly reduced infarct volume, decreased blood-brain barrier (BBB) breakdown, attenuated edema, and improved neurological and motor functions in a dose-dependent manner (30, 100, and 300 mg/kg), while no such effects were observed in mice pretreated with Zengsunsiwu-tang. Immunohistochemical analysis revealed significant increases in ipsilateral occludin and zonula occludens 1 (ZO-1) expression in Shuanghe-tang-pretreated mice, as well as increased AQP4 immunofluorescence.

**Conclusions:**

These results indicate that Shuanghe-tang may protect against brain injury and promote recovery of neurological function following ischemia.

## 1. Introduction

Stroke is among the leading causes of death and long-term disability worldwide, affecting an estimated 15 million people each year. According to a 2002 report by the World Health Organization, approximately 30% of patients with stroke experience long-term sequelae and reduced quality of life due to stroke-related disability [1, 2]. While tissue plasminogen activator (tPA) has been approved by the FDA for the treatment of ischemic injury, tPA treatment must be initiated within 4.5 hours of occlusion [3]. Due to the substantial limitations inherent to tPA treatment, researchers have aimed to identify alternative pharmacological options for combating ischemic disease. Among such options, the pharmaceutical industry has recently begun to investigate the potential of herbs historically utilized in traditional medicine settings [4].

Traditional medicines are composed of natural products, which represent a promising source of new ingredients for the development of conventional medicines. Indeed, researchers have begun to consult the traditional medicine literature in the search for novel therapeutic strategies [5, 6]. Compiled by the Korean royal physician Heo Jun during the 17th century, the *Dongui Bogam* contains records regarding a number of prescriptions and systematic screening methods that have been applied to both experimental and clinical settings [7, 8]. Researchers have suggested that the *Pung* chapter of the *Dongui Bogam* contains valuable information regarding herbal combinations that may aid in the development of novel stroke treatments [9].

A number of pathophysiological events occur following stroke, including energy failure, glutamate excitotoxicity, oxidative stress, leukocyte infiltration, inflammation, breakdown of the blood-brain barrier (BBB), and edema [10]. The BBB maintains the homeostasis of the central nervous system (CNS), which regulates material exchange between the blood and the brain parenchyma [11]. The primary barrier of the BBB is formed by microvascular endothelial cells, which are connected to one another via tight junctions. Research has indicated that specific tight junction proteins are expressed in these regions and that the endothelial cells of the BBB are tightly wrapped by astrocytic endfeet [11, 12]. Following ischemic stroke, the permeability of the BBB increases, allowing circulating immune cells to infiltrate to the brain [13]. In addition, brain edema promotes expansion of the infarct area [14]. Previous studies have revealed that compromised BBB integrity is associated with decreased levels of tight junction proteins such as zonula occludens 1 (ZO-1) and occludin in experimental models of neurological disease [15]. Aquaporin 4 (AQP4) is the most abundant type of water channel and is mainly expressed in the astrocytic endfeet that surround capillaries in the cortex and striatum [16]. While several lines of evidence have suggested that AQP4 plays important roles in maintaining BBB integrity and edema, research regarding the effects of AQP4 on BBB injury and cerebral edema remains controversial [17–19].

Thus, the prevention of BBB breakdown and edema is critical for advancing research regarding treatment options for ischemic stroke. In the present study, we examined the effects of 29 medicinal herbs listed in the *Dongui Bogam* on stroke symptoms in a mouse model photothrombotic cortical ischemia.

## 2. Materials and Methods

### 2.1. Selection of Medicinal Herbs for the Treatment of Stroke

**Figure 1:**
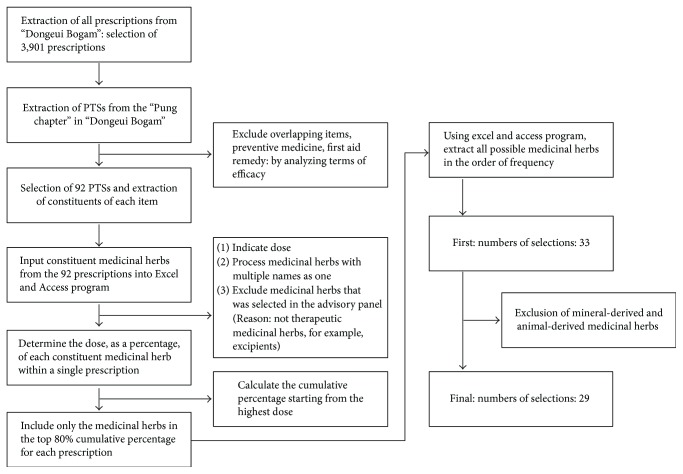
Selection of medicinal herbs for stroke treatment from 92 prescriptions listed in *Dongui Bogam*.

The medicinal herbs examined in the present study were selected by searching all prescriptions for the treatment of stroke (PTSs) listed in the *Pung* chapter of the *Dongui Bogam* via text mining analysis. Among the 92 PTSs identified, we selected medicinal herbs with the highest repeat frequencies in the Korean medical literature (Figure 1). Overlapping combinations of herbs frequently reported in the literature were also chosen as candidates. We then determined the dose of each medicinal herb within a prescription as a percentage of the total prescription. Those with a higher proportion of the medicinal herb were classified as main prescriptions [9]. Only those herbs within the top 80% as a cumulative percentage of each prescription were selected, resulting in a total of 33 herbs. Among these, we further excluded those derived from animals or minerals, resulting in a final total of 29 medicinal herbs (Figure 1). The 29 herbs were extracted via freeze drying, following which the yield of each extract was calculated (Table 1). PKM03-28 and PKM03-29 were excluded from further investigation due to their toxicity and liquid formula, respectively (data not shown).

### 2.2. Preparation of Herb Extracts

The 29 selected herbs were purchased from Hwalim Natural Drug (Busan, Korea). Extracts were prepared by adding an amount of 70% ethanol equal to 10 times the weight of the herb. Each herb was extracted via sonication, which was performed three times for each sample over an interval of 1 h, following which samples were filtered twice and concentrated using an evaporator equipped with a decompression device. The yield of herb extracts was calculated after freeze drying (Supplementary Figure S1A). Extracts of Shuanghe-tang and Zengsunsiwu-tang were by Pusan National University Korean Medicine Hospital. Shuanghe-tang or Zengsunsiwu-tang was boiled in 1.2 L of water for 3 h and then extracted as described above (Supplementary Figure S1B).

### 2.3. Animal Experiments

Male C57BL/6 mice were obtained from Doyeol Biotech (Seoul, Korea). Mice were housed under a 12 : 12 h light/dark cycle and allowed ad libitum access to food and water. All animal protocols were implemented in accordance with Pusan National University guidelines for the care and use of laboratory animals and had been approved by the Institutional Review Board of Pusan National University (PNU-2016-1064). Mice were orally administered 0.15 ml of each herb extract at the appropriate concentration once per day for 2 days, as well as 1 h prior to focal cerebral ischemic injury (total of three treatments). Extracts of Shuanghe-tang or Zengsunsiwu-tang were orally administered to mice once per day for 4 days, as well as 1 h prior to focal cerebral ischemic injury (total of five treatments).

### 2.4. Induction of Focal Cerebral Ischemia

Focal cerebral ischemia was induced via photothrombosis as previously described [20]. Briefly, mice were anesthetized with 2% isoflurane in O_2_ (20%) and N_2_O (80%), following which they received an intraperitoneal (i.p.) injection of rose bengal (Sigma-Aldrich, St. Louis, MO; 0.1 ml of 10 mg/ml in 0.9% saline) 5 min prior to illumination. Mice were fixed on a Stereotaxic Frame (David Kopf Instruments, Tujunga, CA), following which the brain was exposed. A fiber optic bundle containing a KL 1500 LED cold light source (Carl Zeiss, Jena, Germany) was positioned onto the sensorimotor cortex of exposed brain (2.4 mm lateral from the bregma) and illuminated for 15 mins. The scalp was sutured after illumination, at which time the mice were allowed to recover under a heating lamp and returned to their home cages. Body temperature was maintained at 37.5°C during surgery using a heating pad (Harvard Apparatus, Holliston, MA).

### 2.5. Infarct Volume and Edema

Brains were harvested 24 h after ischemic injury, and infarct size was determined via 2,3,5-triphenyltetrazolium chloride (TTC) staining of 2 mm thick brain sections. Infarct size was quantified using i-Solution software (Image & Microscope Technology, Vancouver, Canada). Measurements of direct infarct volume included areas of the ipsilateral side that had sustained direct damage. Indirect infarct volume was calculated according to the following formula: contralateral hemisphere (mm^3^) − undamaged ipsilateral hemisphere (mm^3^). Edema was calculated by subtracting direct infarct volume from indirect infarct volume.

### 2.6. Neurological Score

Neurological deficits were evaluated 24 h after ischemic injury using the following scoring system: 1 = turning in direction of ipsilateral (nondamaged) side when held by the tail; 2 = turning in direction of contralateral (damaged) side and difficulty bearing weight; 3 = unable to bear weight on the contralateral side; 4 = no spontaneous movement [21].

### 2.7. Rota-Rod Test

Locomotor function was examined using the rota-rod test, based on the average latency until falling from the spinning rod (Panlab S.L.U., Barcelona, Spain). Mice underwent pretraining for adaptation trials, following which they were placed on the rotating rod at a speed of 18 rpm for 3 min. Each mouse underwent five trials.

### 2.8. Wire Grip

A wire grip test was used to evaluate the vestibular motor function of mice 24 h after focal cerebral ischemia. Each mouse was suspended on a metal wire and forced to hang using both forepaws. Wire grip was scored as follows: 1 = not holding onto the wire; 2 = holding onto the wire using both forepaws and hindpaws but not the tail; 3 = holding onto the wire using both forepaws and hindpaws as well as the tail, without movement; 4 = moving on the wire using both forepaws, both hindpaws, and tail; and 5 = moving well on the wire.

### 2.9. Determination of Evans Blue Leakage

Evans Blue (2% in saline, 4 ml/kg; Sigma-Aldrich) was injected into the tail vein immediately following photothrombotic ischemia. Twenty-four hours after injection, mice were anesthetized and transcardially perfused with PBS. Brains were removed, following which the cortical area of each hemisphere was separated, weighed, and homogenized in 400 *μ*l of N,N-dimethylformamide (Sigma-Aldrich, St. Louis, MO, USA). Following incubation overnight at 55°C, samples were centrifuged at 13,000 rpm for 20 mins. The absorbance of supernatant was measured at 620 nm via spectrophotometry, while Evans Blue extravasation (*μ*g/g of brain tissue) was quantified using a standard curve [22].

### 2.10. Immunofluorescence Staining for Tight Junction Proteins

Mice were perfused with cold PBS followed by 4% paraformaldehyde 24 h after focal cerebral ischemia. Immediately thereafter, brains were harvested and further fixed for 24 h in 4% paraformaldehyde, following which they were cryoprotected in 30% sucrose for 72 h at 4°C. Each brain was frozen in optical cutting temperature (OCT) compound (Sakura Finetek, Torrance, CA) and stored at −80°C until analysis. The frozen brains were sectioned (thickness: 20 *μ*m) using a CM 3050 cryostat (Leica Microsystems, Wetzlar, Germany). Brain sections were immunostained with anti-ZO-1 (1 : 100), antioccludin (1 : 100, Invitrogen Corporation, Carlsbad, CA), anti-CD-31(1 : 100, BD Bioscience), and anti-AQP4 (1 : 100, Millipore) overnight at 4°C, following which they were incubated with Alexa 488 or Alexa 594-conjugated secondary antibodies (1 : 500, Life Technologies) for 2 h in total darkness. DAPI (molecular probe) was used for nuclei staining. Fluorescence images were captured using a Zeiss LSM 700 laser scanning confocal device (Carl Zeiss, Jena, Germany) and Slide Scanner Axio Scan.Z1 (Carl Zeiss, Jena, Germany). The images are quantified using Metamorph Microscopy Automation and Image Analysis Software (Molecular Devices, USA) and i-Solution software (Image & Microscope Technology, Vancouver, Canada).

### 2.11. Statistical Analysis

Data are represented as the mean ± SEM. Comparisons of mean values between two groups were performed using Student's *t*-tests. The level of statistical significance was set at *p* < 0.05.

## 3. Results

### 3.1. Pretreatment Effects of Medicinal Herbs on Ischemic Brain Injury

**Figure 2:**
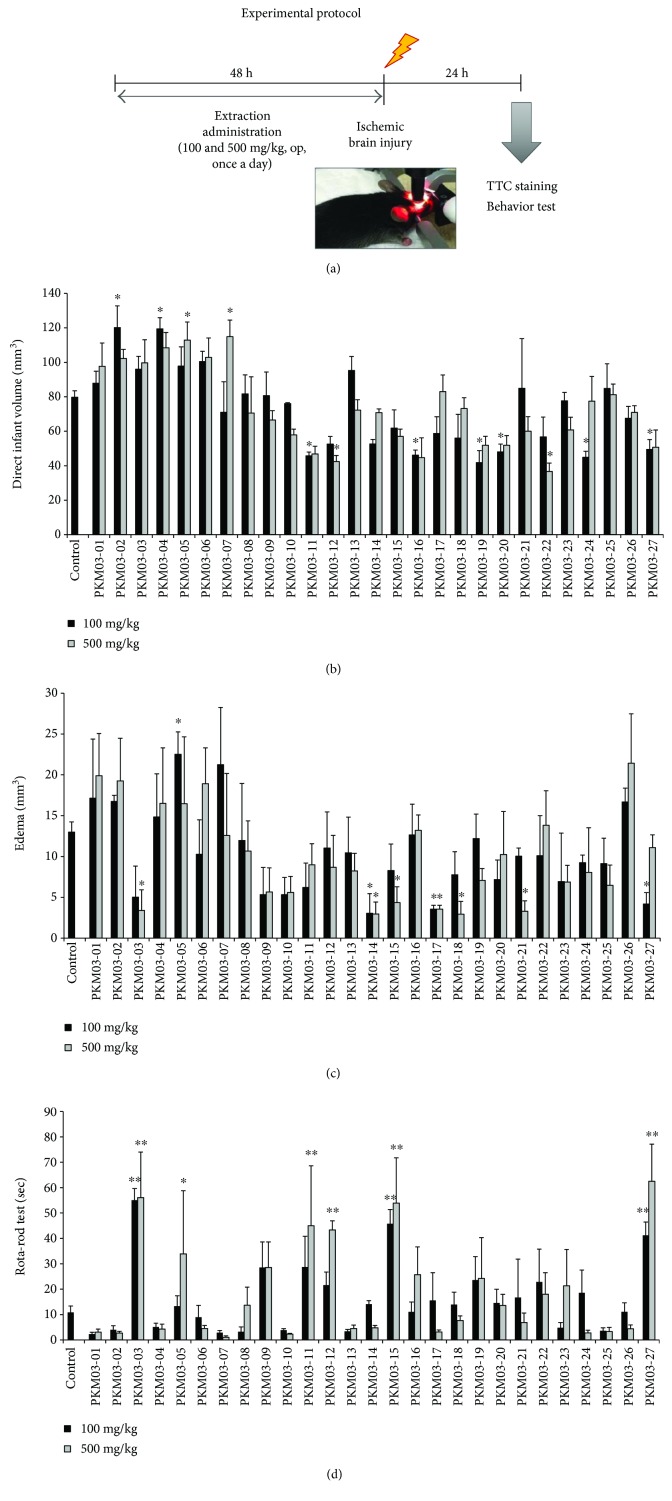
Effects of medicinal herbs on ischemic brain injury following focal cerebral ischemia. (a) Experimental protocol. Mice were pretreated via oral administration of 100 or 500 mg/kg of medicinal herb extracts (*n* = 3 − 4 each) or PBS (control group, *n* = 56) for 2 days prior to focal cerebral ischemia, as well as 1 h prior to the procedure. (b, c) Direct infarct volume (b) and edema (c) were quantified via TTC staining 24 h after injury. ^∗^ *p* < 0.05 versus control group. (d) The rota-rod test was used to examine locomotor function. The average latency time over five trials was recorded for each mouse. ^∗^ *p* < 0.05, ^∗∗^ *p* < 0.01 versus control group.

We evaluated the pretreatment effects of selected herbs on symptoms of focal cerebral ischemia. Mice were orally administered 100 and 500 mg/kg of each extract once a day for 2 days prior to ischemic injury, as well as 1 h prior to the procedure. Twenty-four hours after ischemic insult, the locomotor function of mice was evaluated using the rota-rod test (Figure 2(a)). Our results indicated that 12 herb extracts significantly reduced direct infarct volume (PKM03-02, PKM03-04, PKM03-05, PKM03-07, PKM03-11, PKM03-12, PKM03-16, PKM03-19, PKM03-20, PKM03-22, PKM03-24, and PKM03-27), relative to that observed in the PBS-treated control group (Figure 2(b)). Furthermore, pretreatment with eight candidate herbs (PKM03-03, PKM03-05, PKM03-14, PKM03-15, PKM03-17, PKM03-18, PKM03-21, and PKM03-27) significantly decreased levels of brain edema relative to those observed in the control group (Figure 2(c)). Functional motor outcomes following ischemic injury were significantly improved in mice pretreated with one of the following six candidate herbs: PKM03-03, PKM03-05, PKM03-11, PKM03-12, PKM03-15, and PKM03-27 (Figure 2(d)). Based on our findings, we chose to further examine five herbs demonstrating significant effects in two of the three analyses (infarct volume, edema, and rota-rod test): PKM03-03, PKM03-11, PKM03-12, PKM03-15, and PKM03-27.

### 3.2. Selection of Formulas Containing the Candidate Herbs

Following selection of the five candidate herbs (PKM03-03, PKM03-11, PKM03-12, PKM03-15, and PKM03-27), we searched the traditional Korean medical literature for formulas containing these herbs, based on the following criteria: (1) The candidate herbs are the main components of the traditional Korean medicine formula; (2) the formula includes at least four of the five candidate herbs; (3) the volume of these four herbs comprise over 60% of the total volume of the combined formula. Based on these criteria, we selected Shuanghe-tang and Zengsunsiwu-tang (Table 2). Shuanghe-tang and Zengsunsiwu-tang were extracted via freeze drying, following which we investigated their effects on ischemic injury (Supplementary Figure S1).

### 3.3. Effects of Shuanghe-Tang and Zengsunsiwu-Tang on Ischemic Brain Injury

**Figure 3:**
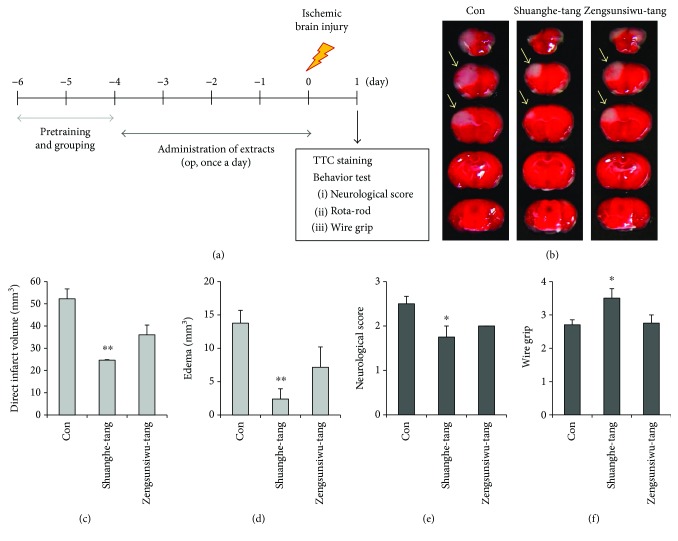
Effects of Shuanghe-tang and Zengsunsiwu-tang on brain function and behavior following ischemic brain injury. (a) Mice were pretreated via oral administration of 500 mg/kg of Shuangh-tang or Zengsunsiwu-tang (*n* = 4) or PBS (control group, *n* = 9) once per day for 4 days prior to ischemic injury, as well as 1 h prior to the procedure. Twenty-four hours after ischemic brain injury, the mouse brains were harvested and stained with 2% TTC solution. (b) Representative photographs of brain sections stained with TTC. White region (arrows) indicates the infarct area. (c, d) Quantification graphs of direct infarct volume (c) and edema (d). ^∗∗^ *p* < 0.01 versus control group. (e, f) Neurological score (e) and wire grip tests (f) were performed to evaluate functional outcomes. ^∗^ *p* < 0.05 versus control group.

Mice underwent oral administration of Shuanghe-tang (500 mg/kg) or Zengunsiweu-tang (500 mg/kg) once per day for 4 days prior to the induction of ischemia, as well as 1 h prior to the procedure (Figure 3(a)). Our findings indicated that oral administration of Shuanghe-tang extract significantly reduced the infarct area (Figure 3(b)), direct infarct volume, and extent of edema, relative to values observed in the control group (Figures 3(c) and 3(d)). Moreover, Shuanghe-tang treatment significantly reduced the severity of neurologic deficits (Figure 3(e)) and improved functional motor ability (Figure 3(f)) relative to findings observed in control mice. In contrast, no significant effects of Zengsunsiwu-tang treatment were observed with regard to infarct volume, edema, neurological deficits, or motor function. We also examined the posttreatment effect of Shuanghe-tang following ischemic injury (Supplementary Figure S2). Posttreatment with Shuanghe-tang did not decrease the level of infarct volume and brain edema relative to the control group. Neurological deficits and functional motor outcomes following ischemic injury were not improved in mice treated with Shuanghe-tang.

### 3.4. Dose-Dependent Effects of Shuanghe-Tang on Ischemic Brain Injury

**Figure 4:**
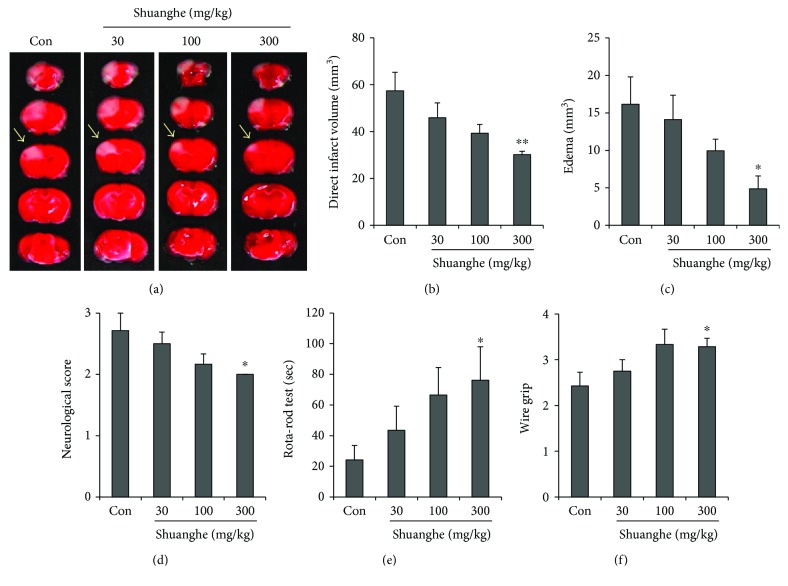
Dose-dependent effects of Shuanghe-tang on ischemic brain injury. Mice were pretreated via oral administration of 30, 100, or 300 mg/kg of Shuanghe-tang (*n* = 6 − 8 each) or PBS (control group, *n* = 7) once per day for 4 days prior to ischemic insult, as well as 1 h prior to the procedure. (a) Representative photographs of coronal brain sections stained with TTC. White region (arrows) indicates the infarct area. (b, c) Quantification of direct infarct volume (b) and edema (c) 24 h postischemia. ^∗^ *p* < 0.05, ^∗∗^ *p* < 0.01 versus control group. (d–f) Neurological score (d), rota-rod (e), and wire grip (f) results were evaluated to assess recovery of neurologic deficit, locomotor function, and vestibular motor function after ischemic injury. ^∗^ *p* < 0.05 versus control group.

To determine whether Shuanghe-tang exerts dose-dependent effects in a mouse model of ischemic brain injury, mice were pretreated with 30, 100, or 300 mg/kg of Shuanghe-tang (Figure 4). Our findings indicated that Shuanghe-tang produced dose-dependent reductions in direct infarct and edema volume. The most significant reductions in infarct volume and edema were observed in mice pretreated with 300 mg/kg of Shuanghe-tang extract (Figures 4(a)–4(c)). We further observed dose-dependent effects of Shuanghe-tang pretreatment on neurologic deficits, locomotor function, and vestibular motor function. Significant improvements in functional outcomes were observed in mice pretreated with 300 mg/kg of Shuanghe-tang extract, relative to those observed in the control group (Figures 4(d)–4(f)).

### 3.5. Effects of Shuanghe-Tang on BBB Disruption

**Figure 5:**
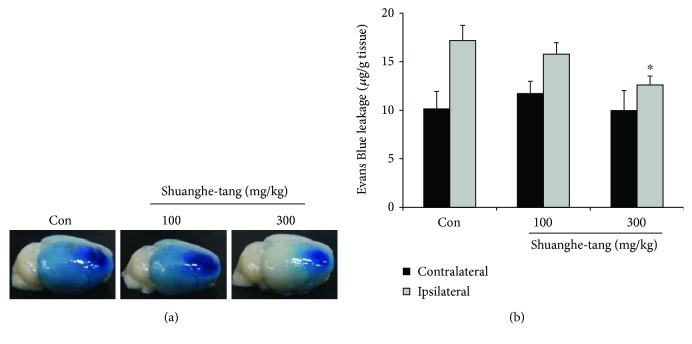
Effects of Shuanghe-tang on BBB disruption after ischemic brain injury. Mice were pretreated via oral administration of 100 or 300 mg/kg of Shuanghe-tang (*n* = 10) or PBS (control group, *n* = 10) once per day for 4 days prior to ischemic insult, as well as 1 h prior to the procedure. Evans Blue (4 mg/kg) was intravenously injected immediately following photothrombotic ischemic insult. (a) Representative photographs of Evans Blue leakage in control or Shunaghe-tang groups 24 h after ischemic injury. (b) Quantification of Evans Blue extravasation. ^∗^ *p* < 0.05 versus control group.

**Figure 6:**
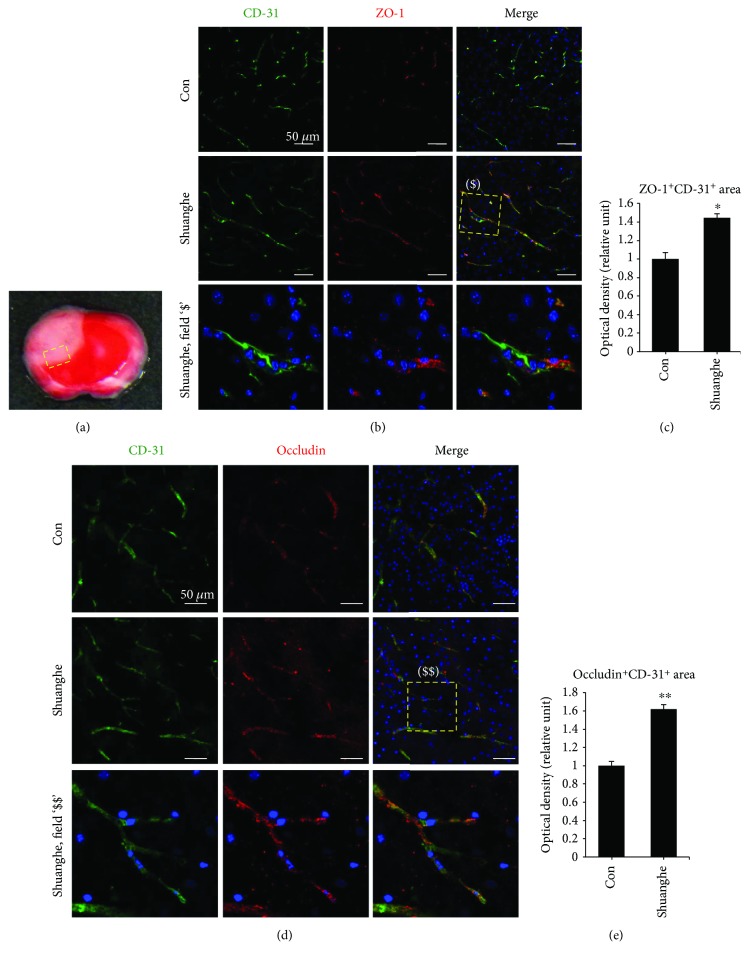
Effects of Shuanghe-tang pretreatment on ZO-1 and occludin levels in ischemic mouse brains. Mice pretreated with Shuanghe-tang (300 mg/kg) exhibited increased expression of the tight junction proteins ZO-1 (b, c) and occludin (d, e) following focal cerebral ischemia. (a) Dashed yellow square indicates the photographed area. Representative photographs of ZO-1 ($) and occludin ($$). CD-31 staining for blood vessels is indicated in green. Scale bar = 50 *μ*m. Quantification graphs of ZO-1 (c) and occludin (e) immunofluorescence (*n* = 6 each, ^∗^ *p* < 0.05, ^∗∗^ *p* < 0.01 versus control group).

Because Shuanghe-tang reduced ischemic brain edema, we evaluated the effect of Shuanghe-tang on the BBB breakdown after ischemia. Evans Blue solution injected to mice extravasates through damaged BBB (Figures 5(a) and 5(b)). After administration of 300 mg/kg Shuanghe-tang, BBB disruption induced by photothrombotic ischemia was significantly reduced, relative to that observed in the control group (Figures 5(a) and 5(b)). We examined the effect of Shuanghe-tang (300 mg/kg) on the tight junction proteins for BBB maintenance (Figure 6). Immunofluorescence staining revealed the increased levels of ZO-1 and occludin even following cerebral ischemia, suggesting that Shuanghe-tang reduces BBB disruption via increases in the level of tight junction proteins.

**Figure 7:**
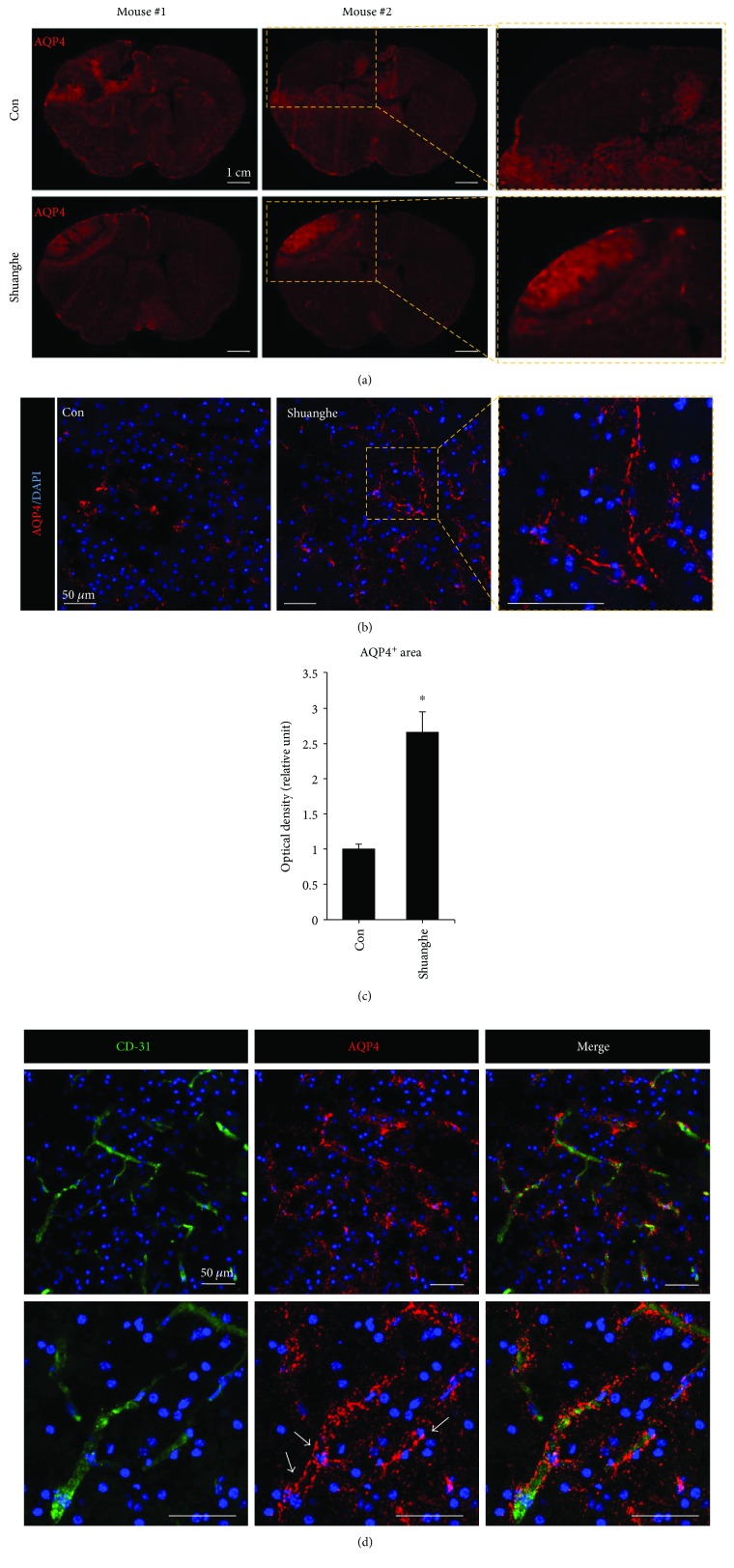
Changes in AQP-4 levels in ischemic mouse brains. (a, b) AQP4 immunofluorescence levels were low in the ischemic area, although such reductions were increased in mice pretreated with Shuanghe-tang. (a) Magnification = ×10; Scale bar = 1 cm. (b) magnification = ×200. Scale bar = 50 *μ*m. (c) Quantification of AQP4^+^ area. (*n* = 3). ^∗^ *p* < 0.05 versus control group. (d) The ipsilateral cerebral cortex of mice pretreated with Shuanghe-tang. AQP4 (red, arrows) restricted to astrocytic endfeet contacts the blood vessel (green CD-31). Scale bar = 50 *μ*m.

We then examined the effects of Shuanghe-tang treatment on levels of the main water channel protein, AQP4. Focal cerebral ischemia showed the low level expression of AQP4 in the peri-infarct region (Figures 7(a)–7(c)). However, mice pretreated with Shuanghe-tang exhibited significant increases in AQP-4 expression in the perivascular region (Figures 7(c) and 7(d)). These findings indicate that AQP4 reduction contributes to BBB disruption and the expansion of brain edema and that Shuanghe-tang extract may protect against secondary injury following ischemia.

## 4. Discussion

In the present study, we evaluated the effects of 27 medicinal herbs selected via text mining analysis of the Korean medical text *Dongui Bogam* in a mouse model of photothrombotic stroke. Based on initial results regarding infarct volume, edema, and rota-rod performance, we further evaluated the effects of five candidate herbs (PKM03-03, PKM03-11, PKM03-12, PKM03-15, and PKM03-27) in our mouse model of ischemic brain injury (Figure 2).

In traditional Korean medicine, *Pueraria lobata* (PKM03-03) has been used to treat fever, headache, and cardiovascular diseases such as hypertension [23], while *Paeonia lactiflora* (PKM03-11) has been used to promote blood circulation and relieve smooth muscle spasms [23]. In addition, *Angelica gigas* (PKM03-12) has been used to treat anemia in women due to its reported effects on hematogenesis [24]. *Cnidium officinale* (PKM03-15) has been used to treat menstrual problems as well as pain associated with rheumatic arthralgia [25]. *Glycyrrhiza glabra* (PKM03-27), one of the most commonly utilized herbs in Korean medicine, has been used in the treatment of renovascular and cardiovascular diseases due to its proven anti-inflammatory, antiulcer, and renoprotective therapeutic potentials [26].

Ancient Korean medical practitioners developed prescriptions using various combinations of medicinal herbs. These combinations of herbs were believed to enhance the therapeutic effect of treatment and reduce toxicity [27]. The medicinal herbs of such prescriptions were classified as follows: Emperor, which was used to refer to the herb with the highest component ratio within the prescription; Minister, which referred to herbs purported to enhance treatment effects; Assistant, which referred to herbs used to reduce the side effects of other ingredients; and Courier, which referred to herbs used to target the desired organ or balance the therapeutic effects of other components [28]. In the present study, we selected two such prescriptions—Shuanghe-tang and Zengsunsiwu-tang (Table 2)—based on the following principles: (1) The candidate herbs are the main medicinal herbs within the prescription (Emperor), (2) the formula includes at least four of the five candidate herbs, and (3) the volume of these four herbs comprises over 60% of the total volume of the combined formula. Shuanghe-tang has long been utilized to treat fatigue and promote recuperation following sickness in Korea [29]. According to the *Dongui Bogam*, Zengsunsiwu-tang has been used in the treatment of continuous bleeding after giving birth [29].

In the present study, mice were pretreated with either Shuanghe-tang or Zengsunsiwu-tang prior to ischemic brain injury (Figures 3 and 4). Our findings indicated that Shuanghe-tang treatment significantly reduced direct infarct and edema volume, in addition to increasing neurological, locomotor, and vestibular function. Such findings indicate that Shuanghe-tang exerts protective effects against ischemic brain damage. In contrast, treatment with Zengsunsiwu-tang produced no significant reductions in the extent or functional impact of ischemic injury.

The BBB is maintained by tight junction proteins (e.g., ZO-1, occludin, and claudin-5) between endothelial cells, astrocyte endfeet, and pericytes [30]. Due to its structure, the BBB plays an important role in maintaining the appropriate concentrations of ions such as Na^+^, K^+^, and Ca^2+^ within a narrow space [31]. Following ischemic stroke, the integrity of the BBB is compromised, and the resulting increase in permeability enables the infiltration of circulating immune cells into the brain [13]. Such infiltration aggravates inflammation and generates neurotoxicity at the damaged site, thereby resulting in neuronal cell death [32]. Therefore, prevention of BBB breakdown represents a major strategy for the attenuation of brain damage following ischemic injury. The results of the present study indicate that Shuanghe-tang pretreatment reduces BBB disruption and increases levels of occludin and ZO-1 in the ischemic brain (Figures 5 and 6).

Alterations in AQP4, a water channel protein that regulates water homeostasis, play an important role in ischemia-induced changes in BBB integrity [16]. It was also reported that AQP4 is associated with neuroinflammation in brain diseases [33, 34]. We observed that downregulated AQP4 in the peri-infarct region was significantly increased in mice pretreated with Shuanghe-tang (Figure 7). We also examined microglia activation in the peri-infarct region using Iba-1 (microglia marker protein) by immunohistochemical staining. But no significant change for Iba-1 was observed in the cortex of Shuanghe-tang pretreatment group relative to the control group (data not shown). These data suggest that neuroinflammation such as microglia activation after ischemic injury was not affect by Shuanghe-tang pretreatment. The effects of AQP4 on BBB injury and cerebral edema remain controversial. Several studies have indicated that AQP4 expression is upregulated in the mouse model of stroke and brain edema could be moderated by downregulation of AQP4 [19, 35]. Conversely, reduction of AQP4 aggravates edema size [18, 36] and Apelin-13 protects against ischemic vascular leakage via AQP4 induction [17]. Taken together, these findings indicate that Shuanghe-tang pretreatment may attenuate BBB dysfunction and edema following ischemic brain injury by promoting increased expression of ZO-1, occludin, and AQP4. However, further studies are required to investigate the mechanisms underlying the effects of Shuanghe-tang on the expression of tight junction proteins and AQP4.

## 5. Conclusion

In the present study, we identified a therapeutic formula for ischemic stroke based on candidate herbs selected via text mining of a traditional Korean medical text (*Dongui Bogam)*. Further analysis in a mouse model of photothrombotic stroke indicated that Shuanghe-tang exerts protective effects against ischemic brain damage and promotes recovery of neurological and motor function following focal cerebral ischemia. Further experimental and clinical investigations of Shuanghe-tang may aid in the development of novel therapeutic strategies for ischemic stroke.

## Figures and Tables

**Table 1:** List of medicinal herbs for the treatment of stroke selected from *Dongui Bogam*.

Family	Scientific name	Portion used	Yield (%)	Sample number
Labiatae	*Schizonepeta tenuifolia* Briquet	Spike	3.34	PKM03-01
Polyporaceae	*Poria cocos* Wolf	Sclerotium	0.434	PKM03-02
Leguminosae	*Pueraria lobata* Ohwi	Root	11.52	PKM03-03
Ephedraceae	*Ephedra sinica* Stapf	Aerial part	3.46	PKM03-04
Rutaceae	*Citrus unshiu* Markovich	Peel	12.88	PKM03-05
Umbelliferae	*Saposhnikovia divaricata* Schiskin	Root	9.66	PKM03-06
Leguminosae	*Astragalus membranaceus* Bunge	Root	12.84	PKM03-07
Ranunculaceae	*Cimicifuga heracleifolia* Komarov	Rhizome	12.34	PKM03-08
Compositae	*Atractylodes lancea* DC	Rhizome	16.08	PKM03-09
Araceae	*Arisaema amurense* Maximowicz	Tuber	2.9	PKM03-10
Paeoniaceae	*Paeonia lactiflora* Pallas	Root	7.713	PKM03-11
Umbelliferae	*Angelica gigas* Nakai	Root	15.013	PKM03-12
Scrophulariaceae	*Rehmannia glutinosa* Libosch. var. *purpurea* Mak	Tuber	20.76	PKM03-13
Umbelliferae	*Ostericum koreanum* Maximowicz	Rhizome, root	12.14	PKM03-14
Umbelliferae	*Cnidium officinale* Makino	Rhizome	9.76	PKM03-15
Lauraceae	*Cinnamomum cassia* Blume	Bark	4.566	PKM03-16
Polygonaceae	*Rheum palmatum* Linne	Rhizome, root	11.77	PKM03-17
Lauraceae	*Lindera strychnifolia* Villars	Root	7.513	PKM03-18
Rutaceae	*Poncirus trifoliata* Rafinesque	Fruit	9.906	PKM03-19
Ranunculaceae	*Aconitum koreanum* Raymond	Tuberous root	19.006	PKM03-20
Campanulaceae	*Platycodon grandiflorum* A. De Candolle	Root	13.33	PKM03-21
Araliaceae	*Aralia continentalis* Kitagawa	Root	7.393	PKM03-22
Araliaceae	*Panax ginseng* C. A. Meyer	Root	10.52	PKM03-23
Zingiberaceae	*Zingiber officinale* Roscoe	Rhizome	7.06	PKM03-24
Orchidaceae	*Gastrodia elata* Blume	Tuber	7.233	PKM03-25
Labiatae	*Scutellaria baicalensis* Georgi	Root	10.606	PKM03-26
Leguminosae	*Glycyrrhiza glabra* Linne	Rhizome, root	16.15	PKM03-27

**Table 2:** Composition of Shuanghe-tang and Zengsunsiwu-tang.

Family	Scientific name	Amount (g)
*Shuanghe-tang*		
Paeoniaceae	*Paeonia lactiflora* Pallas	10
Leguminosae	*Astragalus membranaceus* Bunge	4
Scrophulariaceae	*Rehmannia glutinosa* Liboschitz var. *purpurea* Makino	4
Umbelliferae	*Angelica gigas* Nakai	4
Umbelliferae	*Cnidium officinale* Makino	4
Lauraceae	*Cinnamomum cassia* Blume	3
Leguminosae	*Glycyrrhiza glabra* Linne	3
	Total	32

*Zengsunsiwu-tang*		
Paeoniaceae	*Paeonia lactiflora* Pallas	5
Umbelliferae	*Angelica gigas* Nakai	5
Umbelliferae	*Cnidium officinale* Makino	5
Araliaceae	*Panax ginseng* C. A. Meyer	2
Zingiberaceae	*Zingiber officinale* Roscoe	2
Leguminosae	*Glycyrrhiza glabra* Linne	2
	Total	21

## Supplementary Materials

Supplementary Materials Figure S1: preparation of ethanol extracts (A) and water extracts (B). Figure S2: posttreatment effects of Shuanghe-tang on brain function and behavior in brain injury. (A) After focal ischemic injury, mice underwent oral administration of Shuanghe-tang (300 mg/kg, *n* = 7) or PBS (control group, *n* = 5) once per day for 3 days. Twenty-four hours after final administration, the mouse brains were harvested and stained with 2% TTC solution. (B) Representative photographs of brain sections stained with TTC. White region (arrows) indicates the infarct area. (C, D) Quantification of direct infarct volume (C) and edema (D). (E–G) Neurological score (E), rota-rod (F), and wire grip (G) were evaluated to assess recovery of neurologic deficit, locomotor function, and vestibular motor function after ischemic injury.
