# Supplementary material for: Pretreatment with Shuanghe-Tang Extract Attenuates Postischemic Brain Injury and Edema in a Mouse Model of Stroke: An Analysis of Medicinal Herbs Listed in Dongui Bogam
Source: Oxid Med Cell Longev. 2018 Feb 11;2018:2479602. doi: 10.1155/2018/2479602 (PMC5828342; doi:10.1155/2018/2479602)

Supplementary Figure S1

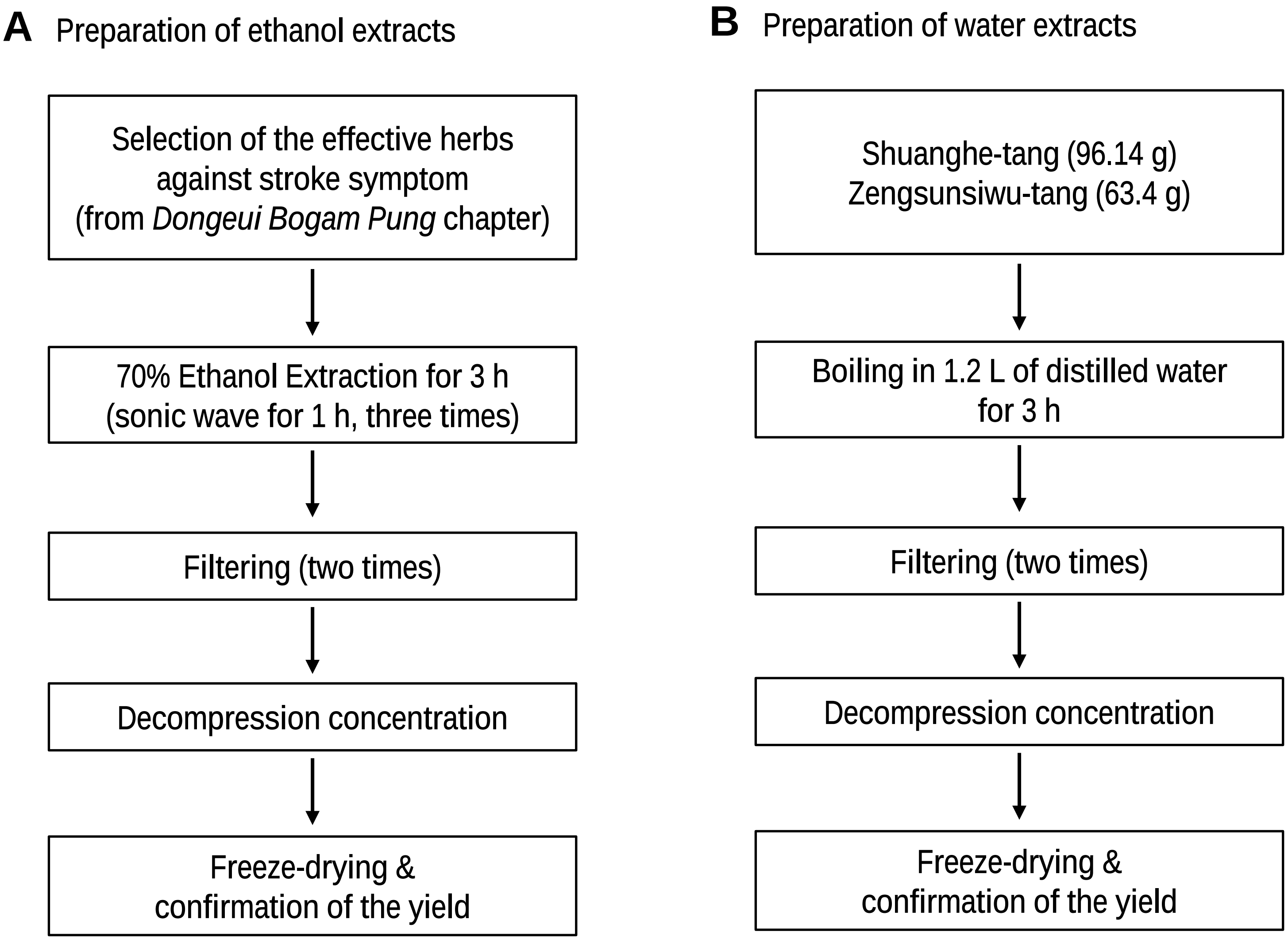

Supplementary Figure S2

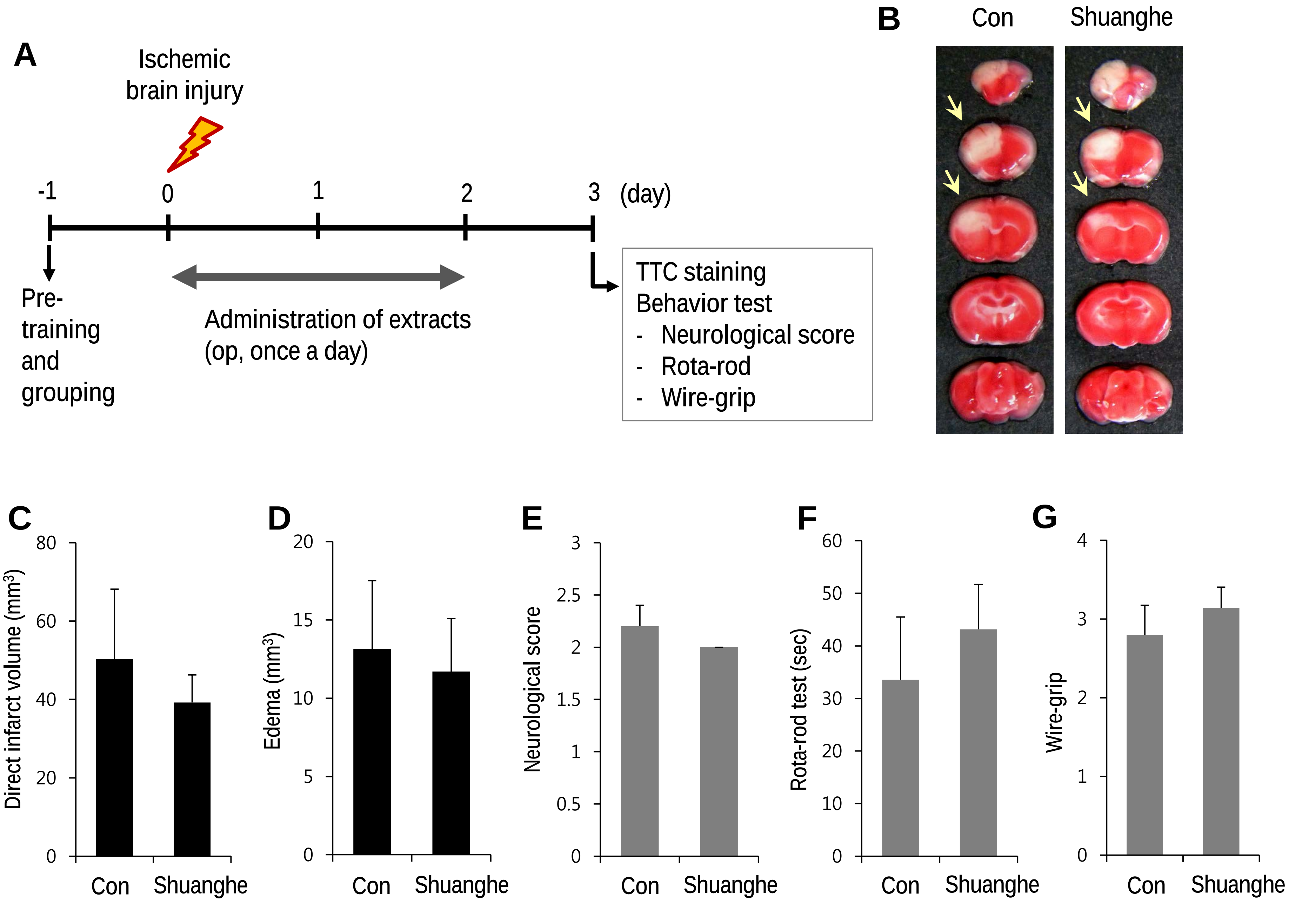

Supplement: Supplementary Materials — Figure S1: preparation of ethanol extracts (A) and water extracts (B). Figure S2: posttreatment effects of Shuanghe-tang on brain function and behavior in brain injury. (A) After focal ischemic injury, mice underwent oral administration of Shuanghe-tang (300 mg/kg, n = 7) or PBS (control group, n = 5) once per day for 3 days. Twenty-four hours after final administration, the mouse brains were harvested and stained with 2% TTC solution. (B) Representative photographs of brain sections stained with TTC. White region (arrows) indicates the infarct area. (C, D) Quantification of direct infarct volume (C) and edema (D). (E–G) Neurological score (E), rota-rod (F), and wire grip (G) were evaluated to assess recovery of neurologic deficit, locomotor function, and vestibular motor function after ischemic injury. [file 2479602.f1.pdf]
